# Editorial: Mucus and the mucociliary interface: continuity and clearance

**DOI:** 10.3389/fphys.2023.1233276

**Published:** 2023-06-13

**Authors:** David B. Hill

**Affiliations:** ^1^ Joint Department of Biomedical Engineering, University of North Carolina at Chapel Hill, Chapel Hill, NC, United States; ^2^ School of Medicine, Marsico Lung Institute, University of North Carolina at Chapel Hill, Chapel Hill, NC, United States; ^3^ Department of Physics and Astronomy, College of Arts and Sciences, University of North Carolina at Chapel Hill, Chapel Hill, NC, United States

**Keywords:** mucus, MUC5B, MUC5AC, cystic fibrosis, mucociliary clearance

During normal respiration, humans inhale thousands of bacteria per hour. The lungs are protected from inhaled particulate and pathogenic material by the biphasic airway surface layer (ASL). Consisting of the mucus and periciliary layers (PCL), the ASL traps foreign matter in the mucus layer, which is swept from the respiratory tract via the action of beating cilia in the PCL. Within the airways, there are regional differences, between the nose and lungs, as well as in the generations of the lung, in the degree of ciliation, percentage of mucin secreting cells, and presence or absence of glands. Muco-obstructive pulmonary diseases (MOPDs), such as cystic fibrosis (CF) and chronic obstructive pulmonary disease (COPD), are characterized by a hyperconcentrated mucus layer that results in increased rates of infection and inflammation, regional differences, and decreased mucociliary clearance (MCC) (Fahy and Dickey, 2010; Hill et al., 2022). The Research Topic of original studies in this Research Topic span a broad selection of investigations into different aspects of the ASL, ranging from the importance of animal and human subjects as model systems for the study of muco-obstructive pulmonary diseases, to experimental techniques designed to bring consistency to biophysical measurements of mucus. The studies collectively close knowledge gaps on regional differences in airway physiology and the role of changing mucus concentration and composition on mucus clearance, as well as highlighting the need for new model systems and standardized measurement methodologies. The development of such standardized techniques and novel model systems is critical to closing knowledge gaps in the pathological alterations MOPDs engender along the airways and to the development of new therapeutic strategies to reduce the enormous public health and economic impact of these diseases.

In general, animal studies offer genetic and environmental control that is impossible to achieve in human studies. Rogers et al. show that animal model systems also allow researchers to characterize regional differences in the airways between the nasopharynx and trachea, such as mucus compositions in the upper *versus* lower airway and mucociliary transport rates. In both murine and rabbit studies, stark differences in mucus clearance rates are shown between the nasal cavities and trachea. While mucus is a nearly continuous sheet in the nose, it is not so in the trachea, where the mucus layer is patchy. The currect study contributes to growing consisences in the literature indicating that the nasopharynx may be better prepared as the first line of defense against inhaled particulates. The Rogers study concludes by showing the utility of animal systems to explore the role of specific genes in airway physiology. The role of *Bpifb1* in pulmonary physiology has proven elusive; however, the present work demonstrates that *Bpifb1* seems to be intricately tied to healthy MCC.

Animal models of CF airway disease have grown in diversity and complexity over the past 30 years (McCarron et al., 2021). The beta ENaC mouse, developed in 2004 (Mall et al., 2004), provided a model system that developed a lung phenotype that was suitable for long-term and longitudinal studies. The recently developed rat model of CF airway disease (Birket et al., 2018) was shown to recapitulate key aspects of CF pathology, including accumulation of mucus in the airways, collapse of the PCL, decreased MCC, and increased mucus viscosity. In the present Research Topic, Keith et al. show that while both Muc5b and Muc5ac (the polymeric gel-forming molecules in mucus) concentrations are increased in the CF rat, the mucus defect is primarily driven by increased Muc5b. Importantly, this study also shows that treatment of G551D CF rats with ivacaftor restores normal mucus function, establishing the CF rat as an effective model system to screen new modulator or genetic therapies.

While the relationship between mucus dysfunction and CF is well documented, there is currently no well-established and agreed-upon protocol to measure the pathological biophysical properties of mucus associated with CF. Völler et al. address this need by examining a host of parameters that may affect the reporting of the rheological properties of mucus and sputum. Given that the physical properties of mucus and sputum are non-linear, i.e., they depend on experimental conditions (Hill et al., 2022), it is critical to tailor not only experimental protocols to match the underlying biology of interest but also to be performed within the linear regime of the physical properties of mucus where data can be generated most consistently. With regard to CF sputum and high-concentration, commercially available bovine sub-maxillary mucin, the Völler et al. study shows that the physical properties of mucus are consistent across wide ranges of applied strains (or deformation). Critically, Völler et al. also show that, for polymeric fluids like mucus and sputum, a solvent trap must be used to limit changes in the concentration of samples during the course of an experiment. When combined with a measurement of the overall concertation of mucus (Hill et al., 2014), the protocol set forth in this study, and its emphasis on the use of solvent traps, will help researchers from different labs faithfully compare results.

Advances in treatment have led to unprecedented improvements in quality of life for people with CF (Barry et al., 2021). However, the benefits to people with CF have resulted in a challenge for researchers. For more than a generation, scientists have been able to collect sputum from CF patients for studies of the nature of mucus and the pathophysiology of CF airway disease, and to test new candidate therapeutic compounds. In the era of highly effective modulator therapy, CF patients are no longer reliably producing sputum, generating the need for new model systems. While mucus may be harvested from cell culture model systems (Hill and Button, 2012), the process is time consuming and expensive. Recently, Markovetz et al. demonstrated that mucus collected from endotracheal tubes (ETT) can serve as a useful model system (Markovetz et al., 2019). In the present Research Topic, Markovetz et al. and colleagues show that isotonic ETT mucus may be pooled and used for biophysical and biochemical characterization of mucus rheology and composition, respectively. They further show that hypertonic ETT mucus, commonly present since patients are not intubated with 100% humidity air, can either be mixed with hypotonic samples or have its tonicity adjusted without compromising the physical properties of mucus. The establishment of the ETT model system provides a cost-effective source of mucus which can help mitigate the loss of CF sputum for research.
